# TWIST1 is a prognostic factor for neoadjuvant chemotherapy for patients with resectable pancreatic cancer: a preliminary study

**DOI:** 10.1007/s00595-023-02655-3

**Published:** 2023-02-10

**Authors:** Sho Fujiwara, Yuriko Saiki, Shinichi Fukushige, Mie Yamanaka, Masaharu Ishida, Fuyuhiko Motoi, Michiaki Unno, Akira Horii

**Affiliations:** 1 Department of Molecular Pathology, Tohoku University School of Medicine, 2-1 Seiryo-machi, Aoba-ku, Sendai, Miyagi 980-8575 Japan; 2 Department of Surgery, Tohoku University Hospital, Sendai, Miyagi 980-8574 Japan; 3 Department of Surgery, Iwate Prefectural Chubu Hospital, Kitakami, Iwate 024-8507 Japan; 4 Department of Surgery I, Yamagata University Graduate School of Medical Science, Yamagata, 990-9585 Japan; 5 Saka General Hospital, Shiogama, Miyagi 985-0024 Japan

**Keywords:** Neoadjuvant chemotherapy, Epithelial-to-mesenchymal transition, TWIST1

## Abstract

Recent advances in the development of chemotherapies have helped improve the prognosis of pancreatic ductal adenocarcinoma (PDAC). However, predicting factors for the outcomes of chemotherapies (either gemcitabine or S-1) have not yet been established. We analyzed the expression of 4 major epithelial-to-mesenchymal transition-inducing transcription factors in 38 PDAC patients who received adjuvant chemotherapy after radical resection to examine the association with patients’ prognoses. The TWIST1-positive group showed a significantly poorer prognosis than the TWIST1-negative group for both the relapse‐free survival (median survival time [MST] of 8.9 vs. 18.5 months, *P* = 0.016) and the overall survival (MST of 15.2 vs. 33.4 months, *P* = 0.023). A multivariate analysis revealed that TWIST1 positivity was an independent prognostic factor for a poor response to adjuvant chemotherapies (hazard ratio 2.61; 95% confidence interval 1.10–6.79; *P* = 0.029). These results suggest that TWIST1 can be utilized as an important poor prognostic factor for radically resected PDAC patients with adjuvant chemotherapy, potentially including neoadjuvant therapy using these agents.

**Supplementary Information:**

The online version contains supplementary material available at 10.1007/s00595-023-02655-3.

## Introduction

Pancreatic ductal adenocarcinoma (PDAC) is a dismal-prognosis tumor with a high mortality rate [1]. The 5-year survival rate is ≤ 10% [2]. Although certain types of neoadjuvant chemotherapy and radiation therapy can improve patients’ outcomes, surgical resection has been the most reliable way to overwhelm PDAC [3, 4]. Recent advances in strategies, including a combination of chemotherapy with conversion surgery for patients with initially unresectable PDAC, have improved the overall survival (OS). However, establishing an ideal method that increases the number of resectable cases after preoperative treatments remains an urgent issue [5–7].

The effectiveness of neoadjuvant and adjuvant chemotherapies with either gemcitabine or S-1 in resected PDAC has been shown by phase II and III trials (Prep-02/JSAP-05 [8] and JASPAC 01 [9], respectively), indicating that gemcitabine and S-1 are important key candidate drugs for overwhelming PDAC. However, which patients can benefit from these drugs remains unclear.

Epithelial-to-mesenchymal transition (EMT) plays an important role in the progression of malignancy in various tumors [10–12]. It has also been shown to be crucial in the acquisition of chemoresistance in PDAC [13]. However, the associations between the expression of EMT-transcription factors (EMT-TFs) and treatment outcomes of gemcitabine or S-1 in the clinical setting remain unclear.

In the present study using pathological R0-resected specimens from PDAC patients who received adjuvant chemotherapy with either gemcitabine or S-1, we investigated the expression of four key major EMT-TFs (SNAIL, SLUG, TWIST1, and ZEB1) by immunohistochemical staining as a preliminary study with the intention of clarifying whether or not EMT-TFs can be prognostic factors for the OS of patients who received gemcitabine or S-1.

## Methods

In this retrospective and stratified cohort study, we analyzed 55 resected specimens from patients with PDAC who underwent microscopically surgical margin-negative resection (pathological R0 resection) at Tohoku University Hospital between January 2007 and June 2012. These specimens were the only tissues available that allowed us to follow the prognoses and conduct immunohistochemical analyses.

Among these 55 patients, 38 received adjuvant chemotherapy, while 17 did not receive adjuvant chemotherapy. The end of the follow-up period was December 2017, with a follow-up rate of 98.2%.

The inclusion criteria were age ≥ 20 years old, histologically proven PDAC, surgical resection with no pathological residual tumor, pathological stages I through III (classified according to the UICC 8th ed.), no distant metastases, and cytologically negative findings for intraoperative peritoneal lavage fluid. The performance status of each patient was adequate (0 or 1) for adjuvant chemotherapy. No radiation therapy was performed in the perioperative period.

The exclusion criteria were PDAC with other pancreatic neoplasms or precancerous lesions, a history of treatment with gemcitabine and/or S-1, confirmed recurrence before starting adjuvant chemotherapy, comorbidity with other malignancy, incompatible general condition for chemotherapy, and an insufficient volume of samples to evaluate four EMT-TFs in consequent slices.

This study was approved by the Ethics Committee of Tohoku University School of Medicine under the accession numbers of 2015-1-473 and 2015-1-474. The primary outcome was the overall survival (OS), and the secondary outcome was the relapse-free survival (RFS).

Patients received the same regimen as in the JASPAC 01 study, a randomized phase III trial of adjuvant chemotherapy with gemcitabine versus S-1 for patients with resected pancreatic cancer, without registration: weekly gemcitabine adjuvant or oral administration of S-1 adjuvant for 6 months [9].

Immunohistochemical staining experiments for EMT-TFs were performed as described previously [14]. The antibodies used were as follows: SNAIL (ab180714; Abcam, Cambridge, UK), SLUG (ab128485; Abcam, or LS-C175177-100; LifeSpan BioSciences, Seattle, WA, USA), TWIST1 (ab50581; Abcam), and ZEB1 (NBP1-05987; Novus Biologicals, Centennial, CO, USA). Heat-mediated antigen retrieval for 30 min by microwaving was performed as follows: 0.1 mol/L citrate buffer for SNAIL and SLUG and 0.001 mol/L EDTA with 0.001 mol/L Tris for TWIST1 and ZEB1. Blocking of endogenous peroxidase activity was performed by incubation in 1% hydrogen peroxidase in methanol for 15 min. Dilutions of primary antibodies were 1:400 (SNAIL), 1:150 (SLUG), 1:400 (TWIST1), and 1:200 (ZEB1), and each antibody was treated with phosphate-buffered saline (PBS) overnight at 4 °C. Immunohistochemical staining with 3,3’-diaminobenzidine were performed for the following durations: 5 min for SNAIL, 10 min for SLUG and TWIST1, and 15 min for ZEB1.

We assessed the entire invasive front of PDAC (× 100), as previous reports suggested that EMT actively occurred in the invasive front [15, 16]. We defined a result as positive if ≥ 10% cells were positively stained to detect differences in the prognosis, considering previous reports; in two previous studies, immunohistochemistry and quantitative reverse transcription polymerase chain reaction (qRT-PCR) showed that almost all resected PDAC samples expressed SNAIL, SLUG, and TWIST1 [11, 14]. We therefore used 10% as the cut-off value according to those previous reports. The expression was quantified and evaluated by a comparison with staining levels of cytoplasm and nuclei in non-cancerous pancreatic duct surrounding PDAC. These analyses were conducted by researchers blinded to the outcome. The expression was reviewed independently by two pathologists who specialized in PDAC.

The chi-square test and Fisher’s exact test were used to categorize variable data. The RFS and OS were analyzed by the Kaplan–Meier method using the log-rank test. The Cox proportional hazards model was used for univariate and multivariate analyses to evaluate the prognostic factors, and those with *P* values below 0.157 were included in the final model considering overfitting [17]. Plausible covariates thought to be associated with prognoses of PDAC and response for chemotherapy were included in a multivariate analysis; adjustment included age, gender, preoperative CA19-9, tumor location, histological differentiation, UICC-stage, UICC T-stage, UICC N-stage, vascular invasion, lymphatic invasion, neural invasion, adjuvant chemotherapy, and expression of TWIST1. Spearman correlation *r* value was calculated to evaluate the correlations of expression of the four EMT-TFs.

Data analyses were performed with the JMP Pro software program, version 14 (JMP, SAS Institute Japan, Tokyo, Japan). Significant differences were considered for a *P* value < 0.05.

## Results

Although previous studies have suggested that EMT plays an important role in chemoresistance and malignant attitudes in various malignant tumors including PDAC [18, 19], the involvement of the expression of EMT-TFs in resected PDAC patients and prognoses has not been fully investigated, particularly in patients who receive adjuvant chemotherapy. We therefore focused on this point in this study.

The patients’ clinicopathological characteristics are summarized in Supplemental Table 1. Of the 55 patients with R0 resection, 38 received adjuvant chemotherapy with either gemcitabine (*n* = 26) or S-1 (*n* = 12) after adequate postoperative recovery according to adjuvant chemotherapy regimens of JASPAC 01. The other 17 patients did not receive adjuvant chemotherapy in the same period. The median follow-up time was 619 days.

In our previous study, we immunohistochemically analyzed the expression of EMT-TFs in epithelial cells in pancreatic intraepithelial neoplasia (PanIN) lesions surrounding the cancerous tissues and reported SNAIL1 as the prognostic indicator [14]. In the present study, we focused on cancerous tissue, particularly at the invasive front of each PDAC. In the 38 patients with chemotherapy, staining was positive for SNAIL in 30 of 38 (78.9%), for SLUG in 30 of 38 (78.9%), for TWIST1 in 18 of 38 (47.4%), and for ZEB1 in 1 of 38 (2.6%). Representative immunostaining results are shown in Supplemental Fig. 1.

To clarify whether or not the expression of the four analyzed EMT-TFs was correlated with each other, we calculated Spearman correlation *r* values. The association between SNAIL and SLUG was moderately positive (*r* = 0.57, *P* < 0.001), but no other correlations were observed.

**Fig. 1:**
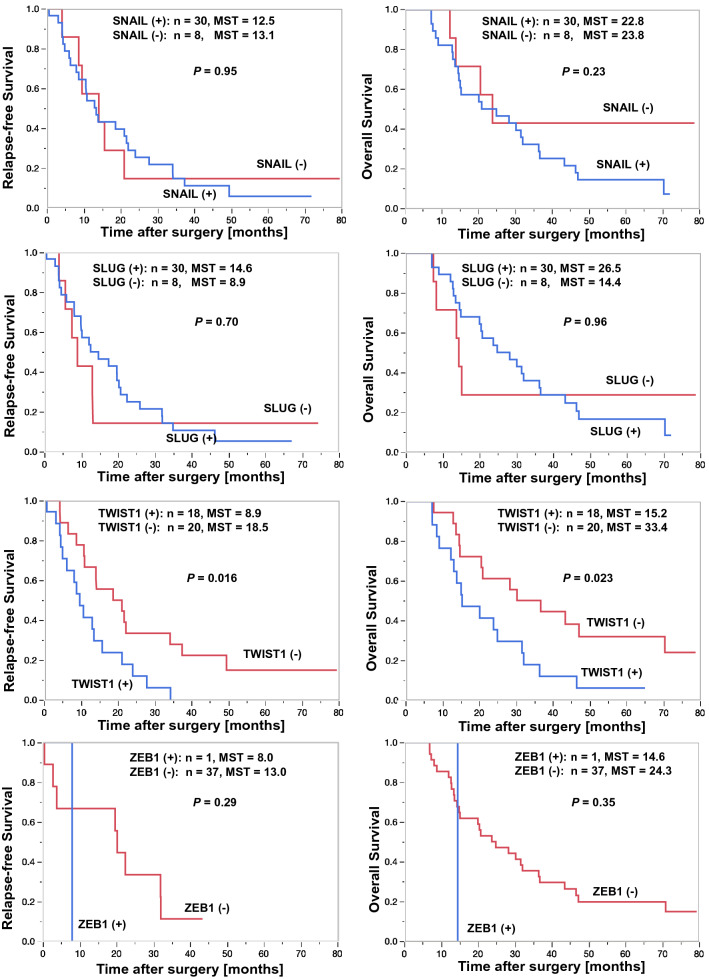
Patients were classified into two groups by the expression of SNAIL, SLUG, and TWIST1. Results of Kaplan–Meier curves for the relapse-free survival (RFS) and overall survival (OS) among patients who received adjuvant chemotherapy are shown. Depending on the TWIST1 expression, significant differences in both the RFS and OS were seen

To identify which EMT-TF contributed most to the prognosis of radically resected PDAC patients with adjuvant chemotherapy, we investigated the associations of the expression of EMT-TFs with patients’ prognoses in the 38 patients who received adjuvant chemotherapies (Fig. 1). The TWIST1-negative group showed a significantly better RFS (median survival time [MST] of 18.5 vs. 8.9 months, *P* = 0.016) and OS (MST of 33.4 vs. 15.2 months, *P* = 0.023) than the TWIST1-positive group. The expression of SNAIL, SLUG, and ZEB1 was not associated with the RFS or OS.

We additionally analyzed the RFS and OS further as shown in Supplemental Fig. 2, including in the 17 patients without adjuvant chemotherapy (A), the 55 total patients irrespective of chemotherapy (B), the 12 patients who received S-1 (C), and the 26 patients who received gemcitabine (D). The results of Kaplan–Meier analyses are shown in Supplemental Fig. 2 (A and B). The TWIST1-positive group with gemcitabine treatment showed a significantly poorer RFS (MST of 9.3 vs. 13.1 months, *P* = 0.049) and a tendency toward poorer OS than the TWIST1-negative group. No other statistically significant differences were observed.

TWIST1 is a transcription factor belonging to a basic helix-loop-helix protein family that cooperates with SNAIL and plays a crucial role in EMT [20]. During the EMT process, TWIST1 is activated by MAPK-mediated phosphorylation [20] and promotes mesenchymal morphologic changes. Inductions of the following molecules by TWIST1 are observed [21, 22]: downregulation of E-cadherin, claudin, occludin, desmoplakin, and plakoglobin; and upregulation of N-cadherin, fibronectin, and vitronectin. These dynamic changes in phenotype play an important role in the malignant attitudes of PDAC. Our results suggest that TWIST1 can contribute to the poor prognosis of PDAC patients receiving adjuvant chemotherapy because of these mechanisms.

To further evaluate TWIST1 as an independent prognostic and surrogate marker for the adjuvant chemotherapy-treated group, we next analyzed whether or not there were any significant associations between TWIST1 positivity and clinical outcome. We found that TWIST1 positivity was associated with an increased UICC N stage but noted no significant associations among any of the other analyzed parameters (Supplemental Table 2). Associations between other EMT-TFs and clinical outcomes are summarized in Supplemental Table 3.

Results of univariate and multivariate analyses are summarized in Table 1. TWIST1 immunostaining (hazard ratio 4.21; 95% confidence interval [CI] 1.84–10.29; *P* < 0.001) and neural invasion (hazard ratio 3.45; 95% CI 1.26–8.72; *P* = 0.018) showed a significant difference with regard to the RFS in the univariate analysis. In the multivariate analysis for the RFS, TWIST1 immunostaining (hazard ratio 4.18; 95% CI 1.79–10.40; *P* < 0.001) showed significant differences. Regarding the OS, gender, adjuvant chemotherapy, and TWIST1 immunostaining showed significant differences, so we further performed multivariate analyses focused on these three parameters and found that TWIST1 immunostaining showed a significant difference with regard to the OS (hazard ratio 2.61; 95% CI 1.10–6.79; *P* = 0.029). These results suggest that TWIST1 is a poor prognostic factor for radically resected PDAC patients with chemotherapy in the clinical setting.

**Table 1:** Univariate and multivariate analyses for the relapse-free and overall survivals

	Univariate analysis for the RFS			Multivariate analysis for the RFS		
	Hazard ratio	95% CI	*P* value	Hazard ratio	95% CI	*P* value
Age (≥ 69 vs. < 69)	0.97	0.48–2.00	0.93			
Gender (male vs. female)	1.81	0.89–3.77	0.10	1.68	0.77–3.66	0.19
Preoperative CA19-9 (> 200.8 vs. ≤ 200.8)	1.41	0.69–2.93	0.34			
Tumor location (pancreatic head vs. body and tail)	0.93	0.41–2.39	0.86			
Histology (poorly vs. others)	1.50	0.44–9.42	0.56			
UICC-stage (stage 3 vs. stage 1, 2)	1.2	0.59–2.60	0.62			
UICC T-stage (T3, 4 vs. T1, 2)	1.1	0.53–2.45	0.80			
UICC N-stage (N2 vs. N0, 1)	1.08	0.49–2.72	0.86			
Vascular invasion (positive vs. negative)	1.4	0.25–3.03	0.60			
Lymphatic invasion (positive vs. negative)	1.18	0.47–2.61	0.70			
Neural invasion (positive vs. negative)	3.45	1.26–8.72	**0.018**	2.25	0.77–6.25	0.13
Adjuvant (S-1 vs. gemcitabine)	0.47	0.20–1.01	0.47			
TWIST1 (positive vs. negative)	4.21	1.84–10.29	**< 0.001**	4.18	1.79–10.40	** < 0.001**

	Univariate analysis for the OS			Multivariate analysis for the OS		
	Hazard ratio	95% CI	*P* value	HR	95% CI	*P* value
Age (≥ 69 vs. < 69)	1.22	0.58–2.63	0.61			
Gender (male vs. female)	2.13	1.01–4.63	**0.047**	2.22	1.13–5.60	**0.023**
Preoperative CA19-9 (> 200.8 vs. ≤ 200.8)	1.28	0.61–2.72	0.52			
Tumor location (pancreatic head vs. body and tail)	0.97	0.43–2.47	0.94			
Histology (poorly vs. others)	1.48	0.44–9.24	0.57			
UICC-stage (stage 3 vs. stage 1, 2)	1.30	0.60–3.03	0.52			
UICC T-stage (T3, 4 vs. T1, 2)	1.17	0.50–2.50	0.71			
UICC N-stage (N2 vs. N0, 1)	1.6	0.65–4.78	0.32			
Vascular invasion (positive vs. negative)	1.95	0.45–5.82	0.33			
Lymphatic invasion (positive vs. negative)	1.39	0.55–3.12	0.46			
Neural invasion (positive vs. negative)	1.61	0.59–3.75	0.33			
Adjuvant (S-1 vs. gemcitabine)	0.17	0.048–0.45	**< 0.001**	0.27	0.069–0.88	**0.029**
TWIST1 (positive vs. negative)	4.19	1.89–9.69	**< 0.001**	2.61	1.10–6.79	**0.029**

*RFS* relapse-free survival, *OS* overall survival, *CA19-9* carbohydrate antigen 19–9, *UICC* Union for International Cancer Control

We also verified the associations between the TWIST1 expression and prognostic impact of chemotherapy using a stratified Cox proportional hazards model. As summarized in Supplemental Table 4, unadjusted and adjusted models showed that TWIST1 positivity was associated with a tendency toward an increased hazard ratio in chemotherapy (adjusted hazard ratio for the OS of TWIST1 positivity: 2.56, 95% CI 0.49–13.5; adjusted hazard ratio for the OS of TWIST1 negativity: 0.48, 95% CI 0.13–1.77).

## Discussion

Various reports in the research setting support our findings, as TWIST1 overexpression has been reported to be a chemoresistant factor in various cancers [22–25]. In terms of chemoresistance during malignant progression, various pathways, such as ABCB1 [23], AURKA [26, 27], COL11A1 [21], and PDGFD [28], have been reported to be involved. Our present findings concerning TWIST1 responses to gemcitabine or S-1 suggest another potential pathway, but the key target molecules remains elusive.

In addition, regarding why SNAIL, SLUG, and ZEB1 were not key molecules affecting the PDAC prognosis, several hypotheses have been proposed. First, the expression of these three molecules was not considered an appropriate prognostic marker. SNAIL and SLUG were expressed in 78.9% of specimens, which was too high. ZEB1 was conversely expressed in just 2.6% of specimens, which was too low. Future studies should explore molecular reasons for these results in greater detail. Second, TWIST1 mainly played a role in stemness and chemoresistance rather than progression and metastasis, although SNAIL and ZEB1 played roles in invasion and metastasis [29]. TWIST1 overexpression also led to an undifferentiated status and the activation of the ras-signaling pathway [29]. These features support TWIST1 as an appropriate for prognostic factor.

In this study using radically resected PDAC specimens from patients with pathological R0 resection, we found an association between a negative expression of TWIST1 and a better prognosis for both the RFS and OS in patients who received adjuvant chemotherapy with either gemcitabine or S-1.

Several limitations associated with the present study warrant mention. First, this was a retrospective study, so the sample size was limited. Although this was an exploratory study, an estimated sample size of 236 patients is required in total to achieve 80% power and 5% significance level as a confirmatory study. However, this sample size would have been too high for us at a single institution to collect a sufficient number of surgical samples to adequately evaluate four EMT-TFs simultaneously. We need to design a multicenter cohort study to overcome this problem in the future. Second, endoscopic ultrasound-guided fine-needle aspiration or a biopsy is required to confirm that the TWIST1 expression is truly a prognostic factor for neoadjuvant chemotherapy. EMT is a complex pathway, and the present study did not reveal details concerning the system of chemoresistance acquisition.

However, despite these limitations, this is the first report investigating the association between these four major EMT-TFs and the prognosis of adjuvant chemotherapy in human PDAC using radically resected human samples with adjuvant chemotherapy. Similar previous research was performed, but it included many cases with advanced-stage disease [30]. Stage III and IV patients accounted for 51% of the population in that study, whereas in our cohort, stage IV patients were excluded, and stage I and II patients accounted for about 80% of our population. Kaplan–Meier plots suggested that TWIST1 did not contribute to the poor prognoses in stage II patients (*n* = 147) but did contribute in all-stage patients (*n* = 177) [31]. These previous findings suggest that the prognostic impact of TWIST1 in advanced-stage PDAC is higher than in early-stage PDAC, which may support the validity of our results and the prognostic impact of TWIST1 in specific patients (i.e., those with radically resected PDAC treated with adjuvant chemotherapy).

Furthermore, our results shown in Supplemental Fig. 2 support our results in Fig. 1 and Table 1, wherein a low TWIST1 expression can aid in screening patients who can benefit from chemotherapy. In addition, we should explore adjuvant therapies other than gemcitabine or S-1 for patients with high TWIST1 levels. We believe that it is worth reporting that TWIST1 can be a candidate surrogate marker for clinical management of patients with PDAC. In future studies, more cases should be accumulated, and more genomic and metabolomic details should be investigated, including samples collected by endoscopic ultrasound-guided fine-needle aspiration or biopsies.

In conclusion, the present findings suggest that the TWIST1 expression is an independent poor prognostic factor in patients with PDAC receiving adjuvant chemotherapy with either gemcitabine or S-1 in the clinical setting. These results may enable us to more effectively select candidate patients likely to benefit from chemotherapy, including neoadjuvant treatments using these agents, and identify possible new targets for treatment among PDAC patients classified into certain subgroups.

## Supplementary Information

Below is the link to the electronic supplementary material.

Supplementary file1 (PDF 287 KB)

Supplementary file2 (PDF 542 KB)

## Data Availability

The datasets analyzed during the current study are available from the corresponding author on reasonable request.
