# Supplementary material for: TWIST1 is a prognostic factor for neoadjuvant chemotherapy for patients with resectable pancreatic cancer: a preliminary study
Source: Surg Today. 2023 Feb 10;53(5):633–9. doi: 10.1007/s00595-023-02655-3 (PMC10110717; doi:10.1007/s00595-023-02655-3)
Supplement: Supplementary file 1 — Supplementary file1 (PDF 287 KB) [file 595_2023_2655_MOESM1_ESM.pdf]

Supplemental Table 1. Characteristics of all analyzed patients irrespective of adjuvant chemotherapy (n=55) , with adjuvant chemotherapy (n=38), and without adjuvant chemotherapy (n=17)

	All patients (n=55)	Patients with adjuvant chemotherapy (n=38)	Patients without adjuvant chemotherapy (n=17)
**Age (years)**			
Median (Range)	69.0 (27-83)	69.0 (27-80)	71.0 (58-83)
**Gender**			
Male/Female	31/24	21/17	10/7
**CA19-9**			
Median (Range)	231.9 (1.6-7440)	200.8 (2.0-7440)	239.9 (1.6-4765)
**Operation procedures**			
Pancreaticoduodenectomy	35	27	8
Distal pancreatectomy	16	10	6
Total pancreatectomy	4	1	3
**Portal vein**			
with reconstruction	20	15	5
without reconstruction	35	23	12
**Residual cancer**			
Positive	0	0	0
Negative	55	38	17
**UICC stage**			
IA	3	2	1
IB	9	5	4
IIA	2	1	1
IIB	30	21	9
III	11	9	2
**UICC T**			
1	10	7	3
2	35	23	12
3	8	6	2
4	2	2	0
**UICC N factor**			
0	14	8	6
1	31	22	9
2	10	8	2
**lymphatic invasion**			
0, 1	17	12	5

2, 3	38	26	12
**Vascular invasion**			
0, 1	6	4	2
2, 3	49	34	15
**Neural invasion**			
0, 1	12	8	4
2, 3	43	30	13
**Adjuvant chemotherapy**			
Gemcitabine	26	26	0
S-1	12	12	0
No adjuvant chemotherapy	17	0	17
**TWIST1 expression**			
Negative	28	20	8
Positive	27	18	9

Supplemental Table 2. Results of TWIST1 immunostaining analyses

	TWIST1		
	**Positive (n=18)**	**Negative (n=20)**	***P* value**
**Age (years)**			
≧69	9	9	1
<69	9	11	
**Gender**			
Male	10	11	1
Female	8	9	
**Tumor location**			
Head	13	15	1
Body and tail	5	5	
**Tumor size**			
≧30mm	11	8	0.33
<30mm	7	12	
**Histology**			
Poorly diff.	1	3	0.61
Others	17	17	
**UICC8th T factor**			
1	3	4	0.63
2	10	13	
3	3	3	
4	2	0	
**UICC8th N factor**			
0	2	6	**0.028**
1	9	13	
2	7	1	
**Anterior serosal invasion**			
Positive	7	5	0.49
Negative	11	15	
**Retroperitoneal invasion**			
Positive	17	16	0.34
Negative	1	4	
**Bile duct invasion**			

Positive	10	8	0.52
Negative	8	12	
**Duodenal invasion**			
Positive	8	8	1
Negative	10	12	
**Portal vein invasion**			
Positive	2	8	0.067
Negative	16	12	
**Artery invasion**			
Positive	2	0	0.22
Negative	16	20	
**Plexus invasion**			
Positive	4	4	1
Negative	14	16	
**Other organ invasions**			
Positive	1	0	0.47
Negative	17	20	
**Gemcitabine/S-1**			
Gemcitabine	12	14	1
S-1	6	6	

Supplemental Table 3. Results of SNAIL, SLUG, and ZEB1 immunostaining analyses

	SNAIL			SLUG			ZEB1		
	**Positive (n=30)**	**Negative (n=8)**	***P* value**	**Positive (n=30)**	**Negative (n=8)**	***P* value**	**Positive (n=1)**	**Negative (n=37)**	***P* value**
**Age (years)**									
≧69	16	2	0.24	16	2	0.24	1	17	0.47
<69	14	6		14	6		0	20	
**Gender**									
Male	17	4	1	16	3	0.71	1	20	1
Female	13	4		14	5		0	17	
**Tumor location**									
Head	21	7	0.65	23	5	0.41	0	28	0.26
Body and tail	9	1		7	3		1	9	
**Tumor size**									
≧30mm	17	2	0.23	15	4	1	0	19	1
<30mm	13	6		15	4		1	18	
**Histology**									
Poorly diff.	2	2	0.19	3	1	1	1	3	0.11
Others	28	6		27	7		0	34	
**UICC T factor**									
1	5	2	0.59	4	3	0.27	0	7	1
2	17	6		18	5		1	22	

3	6	0		6	0		0	6	
4	2	0		2	0		0	2	
**UICC N factor**									
0	6	2	1	6	2	1	0	8	1
1	17	5		17	5		1	21	
2	7	1		7	1		0	8	
**UICC stage**									
1	5	2	0.70	5	2	0.70	0	7	1
2	17	5		17	5		1	21	
3	8	1		1	8		0	9	
**Anterior serosal invasion**									
Positive	23	5	0.081	22	4	0.23	1	25	1
Negative	7	3		8	4		0	12	
**Retroperitoneal invasion**									
Positive	26	7	1	26	7	1	0	33	0.13
Negative	4	1		4	1		1	4	
**Bile duct invasion**									
Positive	14	4	1	16	2	0.24	0	18	1
Negative	16	4		14	6		1	19	
**Duodenal invasion**									
Positive	14	2	0.43	14	2	0.43	1	15	0.42
Negative	16	6		16	6		0	22	
**Portal vein invasion**									

Positive	8	2	1	6	4	0.17	0	10	1
Negative	22	6		24	4		1	27	
**Artery invasion**									
Positive	2	0	1	2	0	1	0	2	1
Negative	28	8		28	8		1	35	
**Plexus invasion**									
Positive	7	1	0.66	4	4	**0.044**	0	8	1
Negative	23	7		26	4		1	29	
**Other organ invasions**									
Positive	1	0	1	1	0	1	0	1	1
Negative	29	8		29	8		1	36	
**Gemcitabine/S-1**									
Gemcitabine	19	7	0.39	18	8	**0.039**	0	26	0.32
S-1	11	1		12	0		1	11	

Supplemental Table 4. Unadjusted and adjusted associations between TWIST1 expression and adjuvant chemotherapies.

	TWIST negative			TWIST1 positive		
	Hazard Ratio	95% CI	*P* value	Hazard Ratio	95% CI	*P* value
**Relapse-free survival**						
Crude	0.77	0.30 - 3.33	0.58	0.97	0.40 - 2.40	0.95
Adjusted[a]	0.69	0.55 - 3.87	0.45	1.04	0.39 - 2.80	0.94
Adjusted[b]	0.78	0.23- 2.66	0.69	2.31	0.55 - 9.62	0.25
**Overall survival**						
Crude	0.99	0.41 - 2.48	0.99	1.16	0.33 - 2.28	0.77
Adjusted[c]	0.92	0.44 - 2.72	0.85	1.11	0.40 - 3.09	0.84
Adjusted[b]	0.48	0.13 - 1.77	0.27	2.56	0.49 - 13.5	0.27

[a]Model 1: adjusted for gender and neural invasion based on Table 1 multivariate analysis for RFS

[b]Model 2: adjusted for age, gender, preoperative CA19-9, tumor location, histology, UICC-stage, UICC T-stage, UICC N-stage, vascular invasion, lymphatic invasion, neural invasion based on Table 1

[c]Model 3: adjusted for gender based on Table 1 multivariate analysis for OS
