# Supplementary material for: TWIST1 is a prognostic factor for neoadjuvant chemotherapy for patients with resectable pancreatic cancer: a preliminary study
Source: Surg Today. 2023 Feb 10;53(5):633–9. doi: 10.1007/s00595-023-02655-3 (PMC10110717; doi:10.1007/s00595-023-02655-3)
Supplement: Supplementary file 2 — Supplementary file2 (PDF 542 KB) [file 595_2023_2655_MOESM2_ESM.pdf]

Supplemental Fig. 1

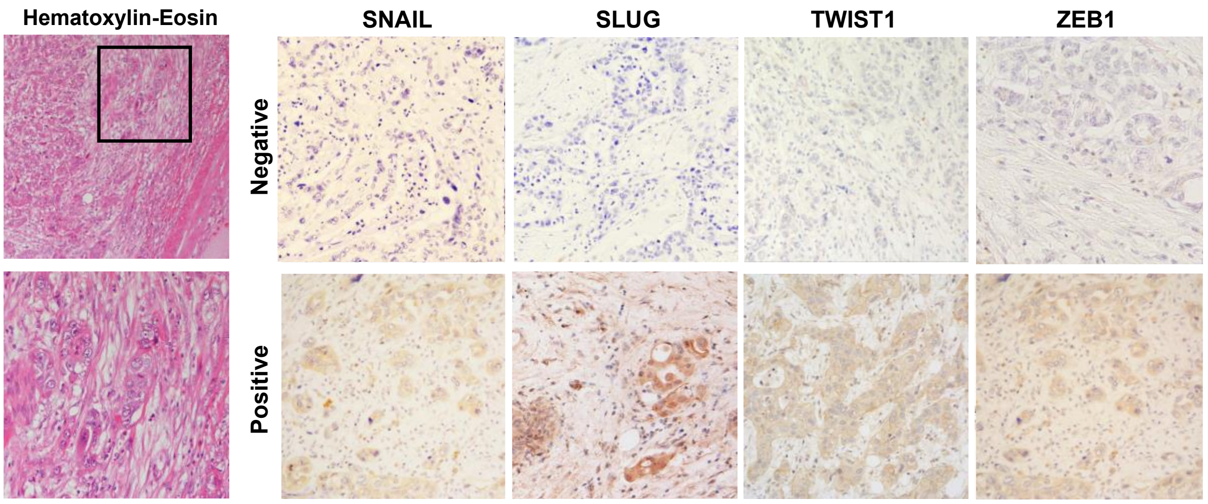

Representative immunostaining results of the four EMT-TFs are shown.

Supplemental Fig. 2

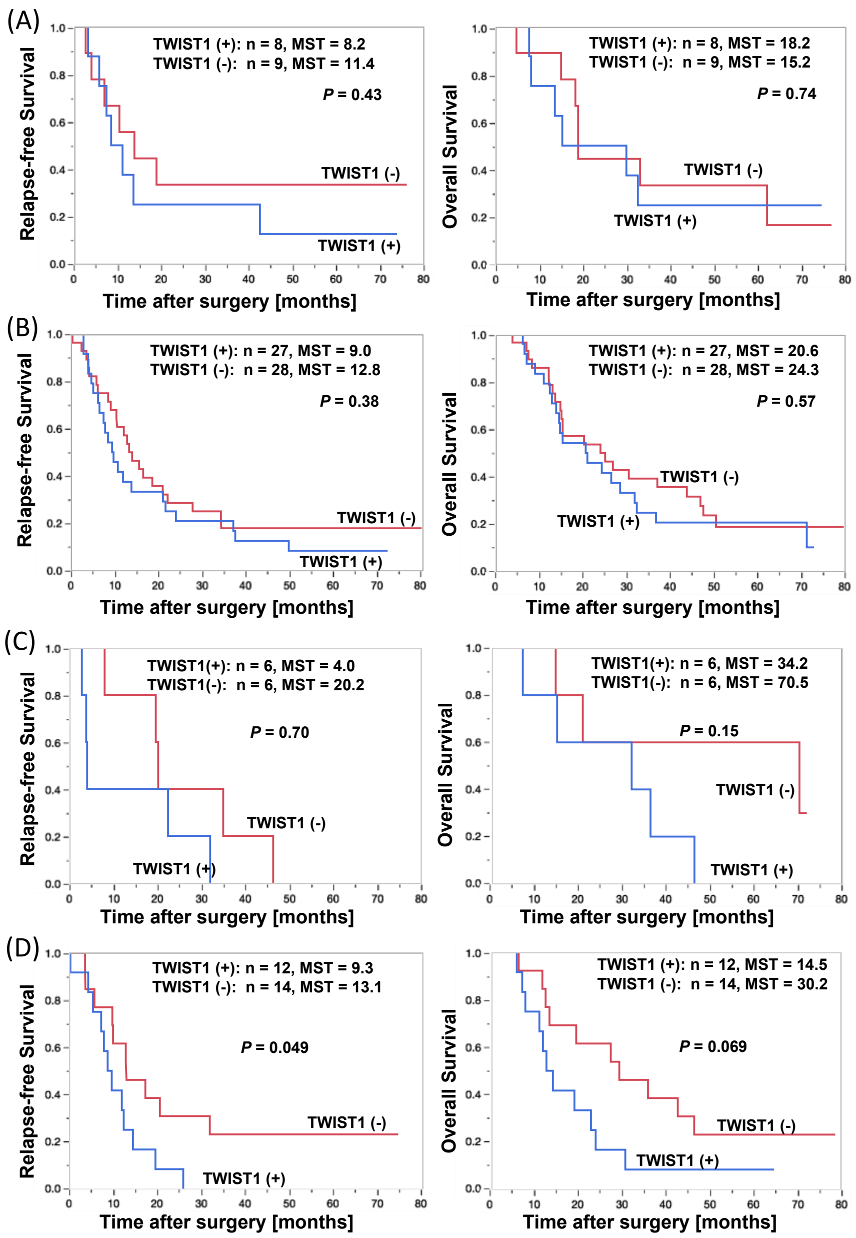

Results of Kaplan-Meier curves for relapse-free survival (RFS) and overall survival (OS) in the 17 patients without adjuvant chemotherapy (A), the total 55 patients irrespective of chemotherapy (B), 12 patients who received S-1 treatment (C), and the 26 patients who received gemcitabine treatment (D).
